# Trained health extension workers correctly identify high blood pressure in rural districts of northwest Ethiopia: a diagnostic accuracy study

**DOI:** 10.1186/s12913-022-07794-w

**Published:** 2022-03-22

**Authors:** Destaw Fetene Teshome, Shitaye Alemu Balcha, Tadesse Awoke Ayele, Asmamaw Atnafu, Mekonnen Sisay, Marye Getnet Asfaw, Getnet Mitike, Kassahun Alemu Gelaye

**Affiliations:** 1 Department of Epidemiology and Biostatistics, Institute of Public Health, College of Medicine and Health Sciences, University of Gondar, Gondar, Ethiopia; 2 Department of Internal Medicine, School of Medicine, College of Medicine and Health Sciences, University of Gondar, Gondar, Ethiopia; 3 Department of Health Systems and Policy, Institute of Public Health, College of Medicine and Health Sciences, University of Gondar, Gondar, Ethiopia; 4 Department of Human Nutrition, Institute of Public Health, College of Medicine and Health Sciences, University of Gondar, Gondar, Ethiopia; 5 Department of Emergency and Critical Care Nursing, School of Nursing, College of Medicine and Health Sciences, University of Gondar, Gondar, Ethiopia; 6 International Institute for Primary Health Care-Ethiopia, Addis Ababa, Ethiopia

**Keywords:** Hypertension, Diagnostic accuracy, Sensitivity, Specificity, Health extension workers, Ethiopia

## Abstract

**Background:**

Hypertension is a public health issue in Ethiopia. The vast majority of cases remain undiagnosed and untreated. Early and accurate identification of hypertension can help with timely management and reduce the risk of complications. In resource-constrained rural settings where poor access to care and a shortage of healthcare providers are major barriers, task-sharing of some primary healthcare duties from well-trained healthcare workers to community health workers has been found to be a cost-effective strategy. This study aimed to assess the ability of trained health extension workers to correctly identify high blood pressure among adults in rural areas of northwest Ethiopia.

**Methods:**

A cross-sectional study was conducted in rural areas of northwest Ethiopia from June to October 2020. Trained health extension workers and health professionals measured the blood pressure of 1177 study participants using a calibrated aneroid sphygmomanometer. A Kappa test statistic was used to compare the two sets of measurements for agreement. The sensitivity, specificity, positive, and negative predictive values were used to assess the validity of health extension workers’ ability to identify high blood pressure in comparison to health professionals.

**Results:**

The trained health extension workers and health professionals identified 219 (18.6%) and 229 (19.5%) of the participants with high blood pressure, respectively. The inter-rater agreement between health extension workers and health professionals for high blood pressure detection was 91.2% (k = 0.912, 95% CI: 0.88, 0.94, *p*-value = 0.000). The sensitivity and specificity of high blood pressure detection by health extension workers were 90.8% (95% CI: 89.6, 92.0) and 98.8% (95% CI: 98.1, 99.5), respectively. While the positive and negative predictive values were 95.0% (95% CI: 92.1, 97.9) and 97.8% (95% CI: 97.3, 98.3), respectively.

**Conclusions:**

The inter-rater agreement between the trained health extension workers and health professionals on high blood pressure detection was excellent. The findings indicate that training health extension workers is a reliable and valid strategy for early detection of hypertension. Thus, the strategy can be integrated with the essential services provided by primary health care units at the village and health post level in rural settings.

## Background

Hypertension is a global public health problem with 1.13 billion adults affected [1]. In Ethiopia, nearly 1 in 4 adults are affected with hypertension [2–5], which is becoming a growing public health problem among rural adults [6–8]. In a nationwide systematic review and meta-analysis conducted in Ethiopia, the pooled prevalence of hypertension was 21.8%, with a slight difference between rural (18.45%) and urban populations (22.85%) [9]. In some settings, the prevalence of hypertension reaches as high as 25% in rural areas [10] and 30 to 35.2% in urban settings [10–14]. Early detection of hypertension is a fundamental step to determine the actual disease burden and initiate appropriate treatment to reduce cardiovascular disease related mortality, limit disability, and improve quality of life [15–18]. However, the vast majority of hypertensive patients remain undiagnosed, untreated or inadequately treated [19] until they have a catastrophic event [20]. According to the 2015 Ethiopian STEPS survey, only 2.8 and 1.5% of hypertensive patients received antihypertensive treatment and controlled their high blood pressure, respectively [21]. The 2018 Ethiopian Noncommunicable Diseases and Injuries commission summary report also showed only 40, 11.2, and 2.9% of patients with hypertension were diagnosed, received antihypertensive treatment, and had their blood pressure controlled, respectively [22].

In Ethiopia, clinicians are primarily responsible for hypertension diagnosis, which presents a significant barrier to early detection and management of hypertension at the primary care level. Ethiopia is one of 57 countries worldwide identified by the World Health Organization (WHO) as experiencing a critical shortage of a health workforce [23]. There were only 0.96 health workforce per 1000 population, which is five times less than the WHO’s minimum threshold of 4.45 per 1000 population [24]. The greatest shortage is for physicians, whose numbers show a decreasing trend at 1: 42,706 population, which is among the lowest ratios in sub-Saharan Africa (2 doctors per 10,000 population) [23]. This is particularly the case for the rural areas of Ethiopia, where more than 80% of the population is inhabited [25].

The human resources gap demands a strategy that aids in the efficient use of available resources. In many developing countries, task-sharing of some primary healthcare activities such as blood pressure measurement, health education, and behavioral counselling interventions from high-level healthcare providers to community health workers (CHWs) has been found to be a cost-effective strategy [26, 27]. This strategy brings diagnostic services closer to the community, saving time and transportation costs for the general population. Several countries have included this intervention into their basic benefits packages and are working to implement it through a primary health care approach [28]. For example, in low and middle income countries, CHWs-led home or community-based hypertension screening programs have been found to be feasible and effective methods of reaching large numbers of previously undiagnosed hypertensive individuals [29], improving clinical linkage to hypertension care [16], and early treatment and blood pressure control [30–33].

However, there is no evidence to suggest that HEWs, key drivers of the health extension program in Ethiopia [34], can correctly identify high blood pressure. And yet, the Ministry of Health Ethiopia has provided a roadmap for optimizing the health extension program, where the packages include prevention and treatment refills for non-communicable diseases, including hypertension [35]. The aim of this study was to assess the ability of trained HEWs to detect high BP or screen for hypertension in adults living in rural districts of northwest Ethiopia. The findings of the study will be used as a starting point for integrating hypertension management at the primary health care level by HEWs through a health system strengthening approach.

## Methods

### Study setting

This study was conducted in rural districts (Dabat and Gondar Zuria) of northwest Ethiopia. Dabat is one of the districts in the North Gondar Zone of the Amhara Regional State of Ethiopia, located about 821 km northwest of Addis Ababa and 75 km from Gondar, a historical place in Ethiopia. Gondar Zuria is one of the districts in the Central Gondar Zone of the Amhara Regional State of Ethiopia. Each district has both urban and rural kebeles (the lowest administrative areas in Ethiopia). Each rural health center has 5 health posts working in a referral system with a primary hospital, forming together a Primary Health Care Unit (PHCU). Each health center with health posts serves 15,000 to 25,000 people [36]. Two to three HEWs are assigned to each health post based on nationally agreed-upon criteria that include residence in the village, ability to speak the local language, completion of 10th or 12th grade, and willingness to serve communities [37, 38]. For a year, all the HEWs received both theoretical and practical training in training institutions [39]. They are government employees who are paid on a regular basis. They worked on 16 packages, which were later expanded to 18 packages in four major categories: hygiene and environmental sanitation, disease prevention and control, family health services, and health education and communication [40].

### Study design and participants

A community-based cross-sectional study design was conducted from June to October, 2020. According to the Ethiopian Central Statistical Agency’s (CSA) population projections for 2014–2017, the total rural population of Dabat and Gondar Zuria districts were 149, 784 and 199, 831, respectively. The study population for hypertension screening was adult population in the rural areas of northwest Ethiopia. All adults aged ≥18 years who have lived in the study area for at least 6 months were eligible for the screening of hypertension. Severely ill patients, mentally disabled, and pregnant women were excluded from the study.

### Sample size determination and sampling method

A total of 1177 people was calculated using a single population proportion formula, with a 14.7% estimated prevalence of hypertension in rural areas [41], a 95% confidence interval, a 3% margin of error, a design effect of 2, and a 10% non-response rate. A multi-stage sampling strategy was used to select the study participants. First, 20 (30%) of the total 72 rural kebeles in the two selected districts were selected using simple random sampling technique. Second, using a simple random sampling technique, 3–4 villages were selected. Finally, one study participant was chosen at random from each household. This validation study included one HEW per kebele.

### Health extension workers (the index test)

According to the districts health office, there were 70 and 102 HEWs in the rural areas of Dabat and Gondar Zuria districts, respectively, in 2018. The health extension workers are all females between the ages of 25 and 35 years, with the majority having more than 10 years of working experience ranging from 5 to15 years. Each HEWs in the chosen district performs community mobilization and health education; promotes and implements hygiene and environmental health; prevents and controls common communicable diseases (HIV/AIDS, malaria, and tuberculosis); provides basic nutrition information to clients; promotes and provides antenatal care, institutional delivery, and postnatal care; promotes and implements immunization and family planning service; and manages childhood illness. A supervisory team of a health officer, nurse, an environmental/hygiene expert, and a health education expert supervises their overall activities.

### Health professionals (the reference standard)

We defined health professionals in this study as those that had a university degree in the field of nursing and public health officer. A total of five health professionals from the University of Gondar were recruited to serve as a reference standard, including two nurses with a Master of Science (MSc) in emergency nursing and three public health officers with a master of public health (MPH) degree. The health professionals have 2 to 8 years of clinical experience and all of them are academicians with at least 4 years of experience at the University. Furthermore, they are actively involved in community service and research.

### Training of health extension workers, health professionals, and supervisors

A three day training was provided for 20 HEWs and 5 health professionals to reduce the amount of variability, a potential source of error. The training covered both theoretical and practical aspects of hypertension and BP measurement procedures. The theoretical session covered hypertensive disease definition, symptoms, risk factors, complications, management and clinical targets, and BP-taking skills. The practical session also covered precautions to take before taking BP, proper body positioning during measurement, and BP measurements. The trainers allowed the trainees to put on the proper cuff size and measure their BP. Each HEW measured three people on average during the practical training. The trainers also observed all of the HEWs prior to their participation in fieldwork to ensure that the systolic and diastolic BP readings were correctly taken and recorded, as well as to identify any performance issues. The training also included information on the questionnaire, interviewing techniques, the purpose of the study, and how to keep the data confidential.

### Blood pressure measurements

The teams (HEWs with health professionals) visited residents’ homes within each selected kebele. They explained the procedure to the participants and obtained verbal informed consent. The HEWs asked participants if they drank caffeinated drinks (tea or coffee) and if they had been working within 30 min or not. It was ensured that they did not consume caffeine, alcohol, or engage in physical activity for at least 30 min prior to the BP measurement. The HEWs instructed the study participants to sit for 5 min before having their BP measured. The HEWs also asked study participants to sit with their back straight, feet flat on the floor, legs uncrossed, and arm supported on their knees with the upper arm at heart level [42]. The HEWs took the first BP measurements at the left arm with an aneroid sphygmomanometer to the nearest 2 mmHg. After a 5 min rest, trained health professionals who were not aware of (blinded to) the HEWs measurement readings independently measured the second BP of the same participant using the same procedure and aneroid sphygmomanometer. The health professionals also took the third measurement 30 min later to arrange for an individual to be referred to a nearby health care facility where care is available.

### Data quality assurance

On a daily basis, the supervisors independently observed and recorded both the HEWs’ and the health professionals’ blood pressure measuring technique using a check list. The checklist included information for taking precautions, deflating the blood pressure cuff before taking blood pressure, uncovering the participants’ left upper arm, placing the blood pressure cuff on the participants’ arm, positioning the stethoscope, inflating the blood pressure to the recommended level, and deflating the blood pressure cuff by 2 mmHg per second. Information exchange by telephone and close supervision by the principal investigator were made daily. The principal investigator of the study also conducts a spot check of the data collection process. The aneroid sphygmomanometers were inspected and calibrated daily against the standard mercury sphygmomanometer to ensure the accuracy of the readings.

### Operational definitions

High blood pressure, both for the index test and the reference standard, was defined as systolic blood pressure **(**SBP**)** ≥ 140 mmHg or diastolic blood pressure **(**DBP) ≥90 mmHg or both. Whereas, individuals with optimal BP was defined as having a SBP of < 140 mmHg and a DBP of < 90 mmHg [43]. Rural household assets were used to calculate family wealth [44], which was then divided into three equal-sized groups (poor, medium, and reach) based on their relative position on the household wealth index.

Reliability refers to the amount of agreement between the results from the screening test under study and those from a reference test. The Kappa statistic measures the level of agreement between two raters (HEWs and health professionals) while accounting for the possibility of the agreement occurring by chance [45, 46]. The kappa statistic has a range of − 1 to + 1, where 0 represents the amount of agreement expected from random chance and 1 represents perfect agreement between the raters. Landis and Koch suggest the following interpretations for intermediate values: values 0.00 as no agreement, 0.01–0.20 as none to slight, 0.21–0.40 as fair, 0.41–0.60 as moderate, 0.61–0.80 as substantial, and 0.81–1.00 as almost perfect agreement [47].

The screening test accuracy of the HEWs in identifying high BP was defined as the ability of the HEWs to distinguish between those who have and those who do not have high BP. Sensitivity was defined as the ability of the HEWs to identify those people who have high BP or the proportion of adults who have had a classification of high BP by the trained health professionals that were correctly identified as high BP by the HEWs. Specificity was defined as the proportion of adults who had a classification of optimal BP by trained health professionals and also classified as having optimal BP by the HEWs. Positive predictive value was defined as the proportion of high BP classified by the HEWs that was also classified as high BP by the health professionals. The negative predictive value was defined as the proportion of optimal blood pressure classified by the HEWs that was also classified as having optimal BP by the health professional classification.

### Data management and analysis

The data were entered into Epidata version 4.6 and analyzed using STATA version 14. The data was checked for missing values, cleaned, coded, recoded, and variables that could be calculated were computed. The data was described using frequency, percentages, means with standard deviations, and median with interquartile range. The findings were presented in the form of text, tables, and graphs. To calculate the HEWs’ hypertension screening test accuracy and reliability, the BP classifications of health extension workers and trained health professionals were cross-tabulated using a 2 by 2 contingency table. The qualitative agreement of BP measurement classification between trained HEWs and trained health professionals was assessed using the Kappa statistic. The sensitivity, specificity, predictive values, and their 95% confidence intervals for the health extension workers BP classifications were calculated. A *p*-value of less than 0.05 was used to determine statistical significance.

## Results

### Participants’ characteristics

A total of 1177 adult population were participated in the selected kebeles. The median age of the participants was 41 (IQR = 30–55) years. Of the participants, 640 (54.4%) were female, 955 (81.1%) were currently married, 808 (68.7%) could not read or write, 1110 (94.3%) were farmers, and 393 (33.4%) were classified as poor (Table 1).

**Table 1:** Sociodemographic characteristics of study participants in northwest Ethiopia, June–October 2020

Variables	Frequency	Percent
**Sex**		
Male	537	45.6
Female	640	54.4
**Age**		
18–24	56	4.8
25–34	294	25.0
35–44	280	23.8
45–54	237	20.1
55–64	144	12.2
≥65	166	14.1
**Religion**		
Orthodox	1167	99.2
Muslim	10	0.8
**Marital status**		
Single	87	7.4
Married	955	81.1
Divorced	46	3.9
Widowed	89	7.6
**Educational status**		
Unable to read and write	808	68.7
Able to read and write	200	17.0
Primary school	113	9.6
High school	42	3.5
College/University	14	1.2
**Occupational status**		
Farmer	1110	94.3
Merchant	7	0.6
Student	41	0.5
Daily laborer	5	0.4
Others	14	1.2
**Wealth index**		
Poor	393	33.4
Medium	392	33.3
Rich	392	33.3

### Blood pressure profile of the study participants

The overall mean SBP and DBP measured by trained HEWs were 116.83 ± 17.38 mmHg and 71.82 ± 11.24 mmHg, respectively. The overall mean SBP and DBP measured by trained health professionals were 117.2 ± 17.82 mmHg and 72.3 ± 11.27 mmHg, respectively. The mean SBP and DBP for individuals having high BP measured by the trained HEWs were 145.26 ± 13.56 mmHg and 88.47 ± 8.74 mmHg, respectively (Table 2).

**Table 2:** Blood pressure profile of the study participants in northwest Ethiopia, June–October 2020

Raters	Blood Pressure Measures					
	SBP, Mean (SD)			DBP Mean (SD)		
	Optimal BP	High BP	Overall	Optimal BP	High BP	Overall
**Health extension workers**	110.33 ±10.12	145.26 ±13.56	116.83 ± 17.38	68.02 ±7.75	88.47 ± 8.74	71.82 ± 11.24
**Health Professionals**	110.49 ±10.30	145.16 ±15.04	117.2 ± 17.82	68.42 ±7.87	88.41 ± 8.67	72.3 ± 11.27

### Blood pressure classification by health extension workers and health professionals

**Fig. 1:**
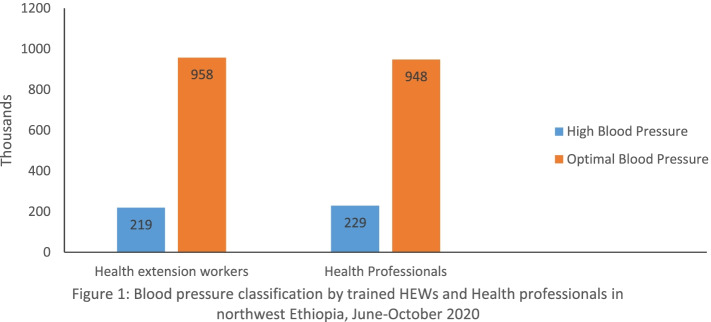
Blood pressure classification by trained HEWs and health professionals in northwest Ethiopia, June–October 2020

Of the total of 1177 adults screened for hypertension, 219 (18.6, 95% CI: 16.4, 20.8) and 229 (19.5, 95% CI: 17.2, 21.7) were identified as having high BP by the trained HEWs and health professionals, respectively (Fig. 1).

### Comparison of the two measurement sets

Of the 219 adults identified as having high BP by HEWs, 208 were also identified as having high BP (true positives) by health professionals, with only 11 classified as having optimal BP (false positives). Of the 958 adults identified as having optimal BP by the HEWs, 937 were also classified as having optimal BP (true negatives) by the health professionals, while 21 were classified as having high BP (false negatives) (Table 3).

**Table 3:** Blood pressure classification of HEWs compared to that of health professionals in northwest Ethiopia, June–October 2020

Trained HEWs	Trained health professionals		Total
	High BP	Optimal BP	
**High BP**	208	11	219
**Optimal BP**	21	937	958
**Total**	229	948	1177

### Percent and kappa measures of agreement

The HEWs and health professionals agreed on 97.28% of the blood pressure measurements readings of the participants. Taking into account chance agreement, the kappa statistic revealed that there was nearly perfect agreement between HEWs and health professionals in correctly identifying high BP. This kappa value is significantly different from zero (Kappa = 0.91, 95% CI: 0.88, 0.94, *p*-value = 0.000) (Table 4).

**Table 4:** Measure of agreement and screening test accuracy of the HEWs blood pressure classification when compared to health professionals in northwest Ethiopia, June–October 2020

Parameters	Value (%) (95% CI)
Percent agreement	97.3 (96.4, 98.2)
Kappa	91.2 (88.0, 94.0)
Sensitivity	90.8 (89.6, 92.0)
Specificity	98.8 (98.1, 99.5)
Positive predictive value	95.0 (92.1, 97.9)
Negative predictive value	97.8 (97.3, 98.3)

### Screening test accuracy of the HEWs in identifying high blood pressure

The sensitivity and specificity of HEWs’ in detecting high BP were 90.8% (95% CI: 89.6, 92.0) and 98.8% (95% CI: 98.1, 99.5), respectively. The health extension workers high BP identification had a 95.0% (95% CI: 92.1, 97.9) positive predictive value. The health extension workers optimal BP identification had a 97.8% (95% CI: 97.3, 98.3) negative predictive value (Table 4).

## Discussion

In this community-based hypertension screening study, inter-rater agreement on hypertension screening was excellent between the trained health extension workers and trained health professionals. The findings indicate that trained health extension workers can detect high BP in their localities using an aneroid sphygmomanometer in a reliable way. The high level of agreement could be attributed to intensive training provided to both the HEWs and health professionals. Another possible explanation for the high level of agreement is the presence of supportive supervision during BP measurements. The kappa value in this study was consistent with studies conducted in India and Nepal, where the Kappa measure of agreement for hypertension screening results between the trained primary care workers (health aids, female community health volunteers) and investigators was 62 to 89% [48], and 94.5% [49], respectively. Our finding was also consistent with those of a study conducted in four developing countries: Bangladesh, Guatemala, Mexico, and South Africa. Those studies found that trained CHWs can conduct primary screening for cardiovascular disease risk, including hypertension, in community settings just as effectively as trained health professionals, with a 96.8% mean level of agreement between the two [50].

The screening test accuracy of HEWs in identifying high BP was found to be excellent in this study, with 90.8% sensitivity and 98.8% specificity. This indicated that a well-trained HEW could identify 9 out of 10 people with high BP. Previous observational and interventional studies in developing countries concluded that CHWs can screen for and detect hypertension. For example, our findings are consistent with a study conducted in Nepal among adults aged 35–74 years for cardiovascular disease risk screening, including hypertension, in which the sensitivity and specificity of the female community health volunteer screening test were 90.3 and 97%, respectively [49]. A study in rural Uganda supported our findings that village health workers effectively screen patients for hypertension in monthly village-based clinics [51]. The ending Eclampsia project in Nigeria showed community health extension workers at primary health care facilities can correctly measure BP, detect high BP, and treat accordingly for severe pre-eclampsia and eclampsia [52]. In Korea, a community-based interventions led by CHWs have shown promising results in early detection of hypertension, linkage to health facilities, improved medication adherence, and BP control [53]. Similarly, a study conducted in low and middle income countries confirmed that CHWs can detect and treat individuals with hypertension and other priority chronic diseases with proper training, supervision and logistical support [54].

This study also found that the accuracy of HEWs’ BP classification for high BP in comparison to a trained health professional’s classification for high BP had an excellent positive predictive value (95.0%) and negative predictive value (97.8%). Thus, once a person is identified as having high BP by the HEWs, the person has a 95% chance of having high BP. Similarly, once an individual was identified by the HEWs as having optimal BP, the individual had a 97.8% chance of having optimal BP. This suggested that HEWs could be taught to use an aneroid sphygmomanometer and stethoscope to screen for hypertension and detect high BP. However, a single blood pressure measurement performed by HEWs is insufficient for diagnosing hypertension and initiating antihypertensive treatment, and should only be used as a screening strategy. Individuals with high BP should be referred to the nearest healthcare facility that provides hypertension care and treatment. In this study, those participants with high BP are therefore referred to the nearest healthcare facility for hypertension diagnosis and treatment.

This study has shed light on a possible task sharing strategy for hypertension screening by health extension workers. However, a single measurement of BP by the HEWs and health professionals may affect the test accuracy. Despite its effect on accuracy, we anticipate that it will have minimal effect on the inter-rater agreement.

## Conclusion

Health extension workers, with adequate training and supervision, can measure blood pressure and detect high BP just as effectively as trained health professionals. The findings suggest that HEWs-led community-based hypertension screening can be used as a strategy to identify high BP among the adult population in the rural areas of Ethiopia. Thus, this strategy can be integrated with the essential services provided by primary health care units at the village and health post levels in rural settings. More research in similar settings is needed to determine the feasibility and scalability of this hypertension screening strategy at the village and health post levels, with subsequent linkage to the nearest health care facility for hypertension diagnosis and management.

## Data Availability

Data will be available from the corresponding author upon request.

## Abbreviations

BP: Blood Pressure
CHW: Community Health Worker
DBP: Diastolic Blood Pressure
HEWs: Health Extension Workers
SBP: Systolic Blood Pressure
WHO: World Health Organization
